# How Do Employees Understand Negative Leadership? The Non-linear Relationship Between Abusive Supervision and Employee Innovation Behavior: Job Performance as a Moderator

**DOI:** 10.3389/fpsyg.2022.867862

**Published:** 2022-06-16

**Authors:** Kuo-Shu Yuan, Tung Ng, Tung-Ju Wu

**Affiliations:** ^1^Department of Human Resources Management, School of Business, Huaqiao University, Quanzhou, China; ^2^School of Business, Huaqiao University, Quanzhou, China; ^3^School of Management, Harbin Institute of Technology, Harbin, China

**Keywords:** abusive supervision, job performance, employee innovation behavior, cognitive view of emotion, non-linear relationship

## Abstract

This study aims to investigate the non-linear relationship between abusive supervision and employee innovation behavior and further examine how job performance moderates this relationship. Two hundred and seventy-six employees across three industries (restaurant service, tourism, and financial service) in China participated in this study and completed the survey at two time points. The results of curve regression show as follows: (1) There exists a non-linear relationship between abusive supervision and innovation behavior, and (2) job performance moderates the non-linear relationship between abusive supervision and employee innovation behavior. For employees who perform well at work, there exists a U-shaped relationship between abusive supervision and innovative behavior. Whereas, for employees with poor job performance, when abusive supervision reaches a certain level, it will promote employee innovation behavior; and the excess of abusive supervision will inhibit employee innovation behavior, showing an inverted U-shaped curve relationship. The finding suggests it is important for managers to understand the stakes arising from abusive supervision. That is, managers should manipulate the right level of abuse supervision to promote employee innovation behavior.

## Introduction

The innovative behavior of employees is a continuous driving force for enterprise development and plays an important role in improving organizational efficiency and enhancing core competitiveness. Thus, it is essential to find out the antecedents that affect employees’ innovative behavior and their mechanism for managerial practice. Previous studies on employee innovation behavior have focused on contextual and individual factors, of which positive leadership behavior is often regarded as the main source (e.g., Burns, 1978; Bass, 1985; Treviño et al., 2003). However, to raise a richer understanding between leadership behavior and employee innovation behavior, it is also important to look at this relationship from a negative perspective (e.g., Lee et al., 2013; Ambrose and Ganegoda, 2020; Fischer et al., 2021; Li et al., 2021). In addition, negative leadership has become the main source of stress faced by employees at work, which has a certain impact on organizational development. As a typical representative of negative leadership in the workplace, abusive supervision has been considered an important factor affecting subordinates’ innovative behavior (Gu et al., 2017; Wang et al., 2019).

Previous studies on the abusive supervision–employees’ innovative behaviors relationship have focused on its negative effects. That is, abusive supervision leads employees to suspect that their supervisors do not respect their contributions and values, prompting subordinates to reduce their reciprocity in the organization to cope with organizational inequality and in turn, weaken the willingness of innovative behaviors (Yang et al., 2019). However, Shen et al. (2020) found that moderate abusive supervision has a promoting effect on employees’ innovative behavior. Based on activation theory, abusive supervision is viewed by subordinates as a threat to social evaluation, stimulating subordinates’ cognition and emotions. The threat of evaluation brought about by abusive supervision, while consuming subordinate resources, may also motivate other resources (Shum et al., 2014). The moderate pressure of abusive supervision on subordinates helps subordinates to actively activate their own cognitive and emotional resources; generate innovative ideas and implement them to improve the evaluation and recognition of supervisors. In addition, when employees perceive different levels of supervisor abuse, their emotional experience will also be different, which impedes or arouses employees’ behavior (Shum et al., 2014; Yu et al., 2018). Therefore, there may be a “non-linear” relationship between abusive supervision and innovative behavior (Yu and Campbell, 2015).

Specifically, Shum et al. (2014) indicated that it is worthwhile to explore the mechanism of the “inverted U-shaped” curve relationship between abusive supervision and employee innovative behavior. For example, after being abused by supervisors, will employees have different innovative behaviors depending on different situations? The leadership substitutes theory suggested that the organizational contextual factors of the alternative leadership have similar influences on job-related variables (Schriesheim, 1997). Of the 14 leadership substitution factors, task completion feedback can effectively predict employees’ organizational commitment and affect individual behavior (Kerr and Jermier, 1978). Job performance is a way of feedback on task completion, and it is also the primary results of the employees’ evaluation concerning performance at workplace (Borman and Motowidlo, 1993). It can become contextual cues, such as subordinates’ judgment of pressure formation and social evaluation, which affects individual cognition and emotion (Van den Bos et al., 2008; Yu et al., 2018). However, previous studies took the job performance of employees as the outcome variable and paid little attention to its moderating effect (e.g., Pierce and Aguinis, 2013).

Supervisors need to achieve the performance standards set by the organization, and take a responsibility to meet the requirements of the organization, whereas the job performance of subordinates affects the salary and promotion of the supervisor to a certain extent. On the other side, when supervisors evaluate employees’ performance, they may become jealous and wary of high-performing employees in order to prevent employees from replacing them (Tse et al., 2013). According to the cognitive theory of emotion, when employees face different organizational situations, they will activate their own cognitive system to evaluate the potential threats caused by the current work situations, and generate corresponding emotions responding to the evaluation results, which will affect their follow-up behavior (Dewe, 1991). That is, when subjected to abusive behavior by supervisors, subordinates will also adjust their self-perceptions and behaviors based on their own work performance and perceived supervisor’s emotion. That is, employees’ job performance is a clue which may have an influence on the relationship between abusive supervision and innovation behavior.

**Figure 1:**
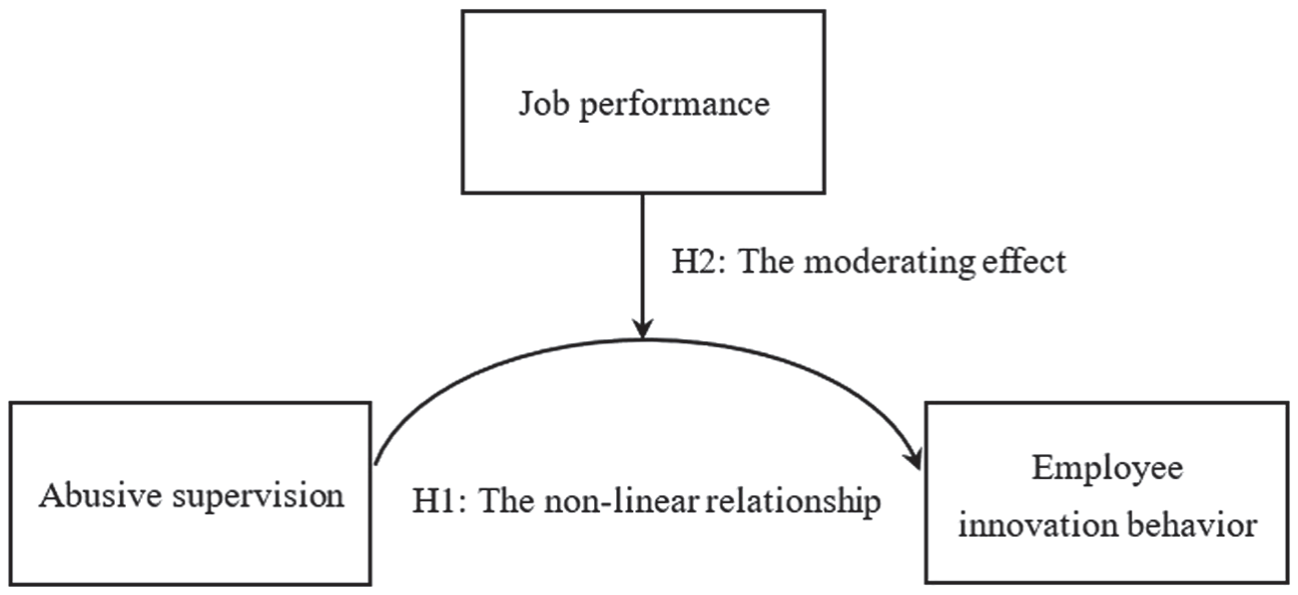
The proposed theoretical model of relationship between abusive supervision, job performance, and employee innovation behavior.

Above all, from the cognitive viewpoint of emotion (activation theory and cognitive theory of emotion), this study aims to examine the boundary conditions of the non-linear relationship between abusive supervision and employee innovative behavior, that is, the moderating effect of job performance (see Figure 1). This study will enrich the field of research on leadership associating with employee innovative behavior.

## Theory and Hypotheses

### Abusive Supervision and Employee Innovation Behavior

Abusive supervision refers to the hostile, non-physical verbal and non-verbal behavior of supervisors toward subordinates for prolonged periods of time (Tepper, 2000). For example, supervisors criticize publicly, vent their anger to subordinates, ignore subordinates’ contributions to the organization, and fail to fulfill their commitments to subordinates. On the other hand, subordinates will regard the abuse of supervisors as a negative stimulus and doubt that the organization does not respect their contributions at all, which reduce their job satisfaction and job performance (Zellars et al., 2002; Lee and Duffy, 2018), cause them to behave against production (Cohen-Charash and Mueller, 2007), and increase their turnover intention (Farh and Chen, 2014; Velez and Neves, 2016). However, not all abusive supervision had a deteriorated effect on the work-related variables. The abuse of a subordinate by a supervisor may not necessarily be out of the intention of harming the employee, but may also be an attempt to urge the subordinate to work more efficiently and perform well under this situation. Previous studies showed that subordinates would still exhibit positive behaviors when they were abused by their supervisors (Ambrose and Ganegoda, 2020).

Chinese traditional Confucian culture inculcates subordinates’ tolerance level to stressors under the negative working situations, such as supervisor’s implementation of humiliation and abuse, which weakens the inhibitory effect of abuse supervision on outcome variables, such as employee innovation behavior (Aryee et al., 2008). Moreover, abusive supervision can help subordinates improve work performance and reduce aggression at work, and moderate level of abusive supervision can even arouse employee creativity (Lee et al., 2013). Specifically, abusive supervision inhibits employees’ innovative behaviors by reducing employees’ psychological safety and promotes employees’ innovative behaviors by increasing challenging stressors (Zhu and Zhang, 2019).

Employee innovation behavior refers to the generation, dissemination, and implementation of novel and useful ideas by employees in the workplace (Scott and Bruce, 1994). It is the self-motivation and self-decision behavior of employees based on a willingness, which is highly unpredictable and risky (Amabile and Pratt, 2016). As the reciprocity principle of social exchange theory describes, employees’ contributions to the organization need to be supported by the organization; if subordinates do not receive authorization and support from their supervisors, their sense of uncertainty in the innovation process will increase, and their willingness to take risks will reduced (Rank et al., 2004). Even for subordinates with high-risk orientation, excessive abusive supervision will gradually weaken their psychological security (Carlson et al., 2011; Yang et al., 2019).

That is, under a high level of abusive supervision, subordinates are not only worried that their own abilities are not sufficient to meet the needs of the organization, but also those high-risk innovative behaviors cannot bring effective feedback to the organization and will be punished, threatening their own development in the organization, and causing subordinates to be unwilling to perform. Loss of engagement further inhibits their enthusiasm and initiative and reduces the occurrence of employees’ innovative behavior (Shen et al., 2020). On the contrary, under a low level of abusive supervision, subordinates perceived less threat of hostile treatment and evaluation, and thus did not form a new stressor (Tse et al., 2013). At this time, subordinates are more inclined to follow the original work style and make less effort at work, which is not conducive to stimulating employees’ innovative behavior.

However, abusive behavior, a source of stress, is one of the ways for supervisors to convey their attitudes and views to subordinates, which can timely let them know that they are not doing well in their work, and then take proactive approaches (Yu et al., 2018). According to activation theory, moderate level of stress is beneficial to activate the neuronal system, stimulate subordinates to take adaptive measures, enhance internal motivation, and engage in work with positive emotions (Shum et al., 2014). Similar, moderate level of abuse can stimulate subordinates to increase their stress, actively adjust their self-cognition and emotional resources in order to gain the approval of their supervisors, and improve their ability to focus on and solve problems (Byron et al., 2010). Subordinates are aware of their own problems, take work responsibility and independent innovation ability, and show their value to the organization, thus promoting their innovative behavior (Wang et al., 2019).

Above all, both high and low levels of abusive supervision may have an adverse effect on employees’ innovative behavior, while moderate abusive supervision can help promote their innovative behavior. Therefore, this study proposes the hypothesis 1:

*Hypothesis 1*: There will be an inverted U-curve relationship between abusive supervision and employee innovation behavior.

### Theoretical Mechanism of Job Performance

Job performance is one of the most concerned work behaviors in the field of management. Traditional research believes that job performance is a general term for employees’ work attitudes and behaviors, mainly reflected in task and contextual performance (Borman and Motowidlo, 1993; Aryee et al., 2007). Chang and Chen (2011) indicated that the performance of employees at work refers to the work quality and workload measured by the supervisors using the key performance indicators of subordinates including work dedication, interpersonal relationship, and task performance (Lindebaum, 2013). It can be seen that although the dimensions of employees’ job performance are different, most of the studies are mainly carried out from the aspects of employees’ task performance, relationship performance, organizational citizenship behavior, etc., reflecting the objective and subjective part of work performances.

Moreover, previous studies often use job performance as an outcome variable to investigate, but ignore the impact of employee performance on supervisors’ emotions, attitudes, and behaviors (Lee and Duffy, 2018). The performance of the supervisors depends on the subordinates to better accomplish the organizational tasks and goals. Nevertheless, high-performing employees are more likely to be envied by other individuals, while they have higher job involvement and are also more likely to acquired organizational rewards and have better career development opportunities (Yang et al., 2019; Chen et al., 2021). This greatly weakens the supervisor’s sense of control over the subordinates, increases their sense of job crisis, and then produces jealousy to the subordinates who perform well (Lee and Duffy, 2018). On the contrary, Low-performing employees lack the necessary job skills, and the quality of tasks performed is substandard. Supervisors have to spend extra working hours to deal with problems brought by these employees, which increases the work burden of the supervisors and causes the supervisor to have negative emotions, such as anger toward these subordinates (Mitchell and Ambrose, 2012; Wang et al., 2018; Rice et al., 2020).

In the face of different emotional displays of supervisors, employees will conduct primary and secondary evaluations (Reisenzein, 1983). According to the cognitive theory of emotion, employees initially evaluate the relationship between the supervisor’s emotional events and emotional response—the situation of employees’ performance evaluation, and generate positive or negative emotions. Then, they attempt to interpret supervisors’ emotional displays to further guide their behaviors (Moors, 2013; Huang et al., 2020). For subordinates who are subjected to abusive supervision, their evaluation of their supervisors’ emotions and behaviors will lead to different responses (Bunk and Magley, 2013). That is, subordinates who perform well at work will not let their supervisor’s treatments dominate their own behavior, but re-evaluate their supervisor’s emotions and behaviors, and control and regulate themselves to produce appropriate behavioral responses (Ambrose and Ganegoda, 2020).

Therefore, the study aims to examine the boundary conditions of relationship between abusive supervision and employee innovation behavior and considers that abusive supervision is regarded by subordinates as a clue of emotional displays of supervisors. That is, subordinates with different levels of performance have different interpretation of abusive supervision, which affects their innovative behavior. In the following section, the moderating role of job performance on this relationship will be explained.

### Moderator of Job Performance

Based on the cognitive theory of emotion (Moors, 2013), in the face of moderate abusive supervision, subordinates with high job performance did not engage in more innovative behaviors than those with low job performance. High-performing subordinates were more confident and optimistic, which can appropriately control the negative emotional experience brought about by their supervisors’ abuse (Ambrose and Ganegoda, 2020; Peltokorpi and Ramaswami, 2021). However, as the supervisors’ abuse deepens to moderate levels, well-behaved employees realize that even greater involvement in innovation will not be rewarded due to supervisors’ jealousy and injustice (Eisenberger, 1992; Yang et al., 2020).

For subordinates with low job performance, the moderate level of abusive supervision can help increase their vigilance and promote more innovative behaviors. Low-performing subordinates face the risk of being marginalized or even fired from the organization, and thus employees are more concerned about staying in the organization than supervisors’ abuse (Rice et al., 2020; Yuan et al., 2020). Based on the cognitive theory of emotion (Moors, 2013), as supervisor’s abuse is assessed as a result of poor performance, subordinates will try to generate naive idea and make breakthrough performances in order to maintain their self-esteem and identity, and stay in the organization (Mitchell and Ambrose, 2012). Therefore, the moderate level of abusive supervision, similar to challenging pressure, has a positive effect on employee innovation behavior (Baer and Oldham, 2006).

Moreover, although the high level of abusive supervision inhibits employees’ innovative behavior, high-performing employees can improve the negative relationship. This is because they have sufficient competency and psychological resources to cope with high level of stress in the workplace, which reduces the uncertainty of innovation and improve the possibility of innovation (Amabile, 1997). In addition, Lim and Lee (2011) indicated that if subordinates used internal attribution to assess that their bosses treated them unkindly and unfairly out of jealousy, they experienced more negative emotions and less satisfaction with their bosses after the primary evaluation. Further, in the second evaluation, they will work harder, come up with creative solutions, and take advantage of opportunities to try to cope with this situation through promotions (Schuler, 1980; Baer and Oldham, 2006). On the contrary, low-performing employees suffer more heavier psychological burden, which is more likely to lead to interpersonal injury behaviors in the workplace. To cope with these injuries and job stress, they have to spend more energy, and then exhaust their work enthusiasm and engagement (Byron et al., 2010). Therefore, the impact of abusive supervision on employee innovation behavior will be positive for subordinates with high job performance, and this relationship will be negative for those with low job performance.

Finally, if the supervisors’ responses meet the expectations of the subordinates, the subordinates will not spend extra effort at work to deal with the cognitive dissonance caused by the supervisors (Carlson et al., 2011). That is, with a low level of abusive supervision, subordinates’ psychological burden and insecurity are at a lower level. For subordinates with high job performance, their sense of superiority reaches beyond the negative emotional experience of abusing supervision, enabling employees to complete work tasks with confidence and easily implement innovation (Zhu and Zhang, 2019). However, for those with low job performance, considering that their abilities are at a low level and innovative behavior is accompanied by high risks, they are not willing enough to perform high-risk tasks, affecting their own interpersonal harmony in the organization and reducing active behavior to innovate (Tse et al., 2013; Lee and Duffy, 2018). This shows that the low level of abusive supervision has a strong inhibitory effect on poor performers to engage in innovative behavior compared to good performers. Based on the cognitive theory of emotion, this study proposes the following hypothesis:

*Hypothesis 2*: Job performance will moderate the curvilinear relationship between abusive supervision and employee innovation behavior. When employees have a lower level of performance, there will be an inverted U-curve relationship. Whereas, when those have a higher level of performance, there will be a U-curve relationship.

## Materials and Methods

### Participants and Procedures

This study investigated the frontline employees of restaurant service, tourism, and financial service in China. The questionnaire was distributed to participants and their supervisors at two time points and over a period of 2 months to avoid method bias. At time 1, supervisors and subordinates provided demographic information and subordinates completed a measure of abusive supervision. One months later, at time 2 supervisors completed measures of employees’ job performance and innovation behavior. Of the 353 matched questionnaires distributed, 312 were returned and 276 were valid, yielding a valid response rate of 78.2%. The valid samples were mostly from women (67.0%); 87.0% of the respondents had a higher level of education; the average respondent age was 29.34 years (*σ* = 8.55) and the average length of respondent employment was 5.77 years (*σ* = 7.83) and the average tenure working with supervisor was 2.51 years (*σ* = 3.62).

### Measures

All measures of study variables used in this study have been developed in previous research. To design a Chinese version questionnaire, the study followed the translation and back-translation procedures to ensure all translated texts are appropriate for investigating the work-related attitudes and behaviors of service industry employees (Brislin, 1980).

### Abusive Supervision

The measure of abusive supervision was developed by Aryee et al. (2008). The ten-item scale using five-point response format (from 1 = never to 5 = always) is adapted to score to match the Chinese context. Sample items are “My supervisor tells me my thoughts or feelings are stupid” and “My supervisor puts me down in front of others.” Cronbach’s *α* value for this scale is 0.894.

### Job Performance

The measure of job performance is from Ingold et al. (2015) task performance scale. The five-item scale with a five-point response format (from 1 = strongly disagree to 5 = strongly agree) for supervisor evaluation was development. Sample items are “The employee demonstrates expertise in all job-related tasks” and “The employee fulfills all the requirements of the job.” Cronbach’s α value for this scale is 0.846.

### Employee Innovation Behavior

The Scott and Bruce’s (1994) innovative behavior scale is used to measure employee innovation behavior in the workplace. The six-item scale with a five-point response format (from 1 = strongly disagree to 5 = strongly agree) for supervisor evaluation is developed. Sample items are “The subordinate promotes and champions ideas to others” and “The subordinate search out new technologies, processes, techniques, and/or product ideas.” Cronbach’s *α* value for this scale is 0.892.

### Control Variables

We controlled for gender, job position, length of employment, tenure working with supervisors, and negative emotion (10-item negative emotion scale from Watson et al., 1988) due to their established relationships with the study variables and in order to be consistent with previous studies. These control variables selected in our study have been found to be significantly related to employee innovation behavior (e.g., Scott and Bruce, 1994; Janssen, 2000) and have been statistically controlled in several studies on abusive supervision (Aryee et al., 2007). Age was measured in years.

### Analytical Strategy

Curve regression models of abusive supervision and job performance on employee innovation behavior are developed to examine study Hypothesis 1 and 2 using hierarchical regression modelling (see Table 1). First, the control variables of gender, job position, length of employment, tenure working with supervisors, and negative emotion are entered into the models with the dependent variables of employee innovation behavior. Then, the main effects of abusive supervision and job performance are entered into the model. In subsequent step, to decrease potential multicollinearity, abusive supervision is mean-centered before the quadratic term set (Aiken and West, 1991). Third, the quadratic term of abusive supervision is entered into the model to examine Hypothesis 1 of the inverted U-curve relationship; the product term of quadratic term of abusive supervision and job performance is also entered into the model to examine Hypothesis 2. Finally, If the regression coefficient of quadratic term of abusive supervision and job performance was significant, the moderating effect was further illustrated to examine the study hypothesis.

**Table 1:** Results of curve regression analysis.

Variables	Model 1		Model 2		Model 3		Model 4		Model 5		Model 6	
	*β*	S.E	*β*	S.E	*β*	S.E	*β*	S.E	*β*	S.E	*β*	S.E
Gender	0.047	0.065	0.045	0.064	0.045	0.063	0.051	0.063	0.051	0.064	0.050	0.062
Job position	0.012	0.089	0.012	0.089	0.015	0.089	0.015	0.095	0.017	0.095	0.017	0.093
Length of employment	0.057	0.006	0.065	0.006	0.072	0.006	0.076	0.006	0.071	0.006	0.105^*^	0.006
Tenure working with supervisors	0.001	0.013	0.003	0.013	0.008	0.013	0.013	0.013	0.017	0.013	0.006	0.013
negative emotion	0.100	0.060	0.093	0.062	0.084	0.057	0.087	0.057	0.090	0.055	0.090	0.056
Abusive supervision			−0.156^*^	0.016	−0.161^*^	0.012	−0.163^*^	0.012	−0.159^*^	0.012	−0.149^*^	0.010
Abusive supervision^2^					0.270^**^	0.027	0.270^**^	0.027	0.160^*^	0.027	0.164^*^	0.026
Job performance							−0.030	0.044	−0.031	0.044	−0.039	0.043
Abusive supervision × Job performance									−0.166	0.148	−0.182^*^	0.077
Abusive supervision^2^ × Job performance											0.338^**^	0.081
*R* ^2^	0.003		0.015		0.034		0.035		0.045		0.073	
∆R^2^			0.012^*^		0.019^**^		0.001		0.010^*^		0.028^**^	

n = 276; ^*^p < 0.050 and ^**^p < 0.010. Dependent variable is employee innovation behavior.

## Results

### Descriptive Statistics and Correlations

Table 2 shows descriptive statistics and inter-correlations among the study variables. Abusive supervision is negatively related to job performance (*r* = −0.160, *p* < 0.010), but not significantly related to innovation behavior (*r* = −0.056, *p* > 0.050). Job performance is positively related to innovation behavior (*r* = 0.181, *p* < 0.050). These control variables (job position, length of employment, tenure working with a supervisor, and negative emotion) are significantly related to abusive supervision, job performance, and innovation behavior and thus we include them in subsequent analyses and reporting of results (Becker, 2005).

**Table 2:** Descriptive statistics and correlations among study variables.

Variables	M	SD	1	2	3	4	5	6	7	8
1. Gender	0.33	0.47	–							
2. Job position	0.28	0.20	0.043	–						
3. Length of employment	5.77	7.83	−0.132	0.275^***^	–					
4. Tenure working with supervisions	2.51	3.62	−0.056	0.237^***^	0.569^***^	–				
5. Negative emotion	2.34	0.91	−0.206^**^	0.109	0.136	144	(0.920)			
6. Abusive supervision	1.51	0.59	−0.113	0.059	0.168^*^	0.153^*^	0.312^***^	(0.890)		
7. Job performance	3.26	1.02	−0.127	0.320^***^	0.170^*^	0.309^***^	−0.160^*^	0.037	(0.846)	
8. Innovation behavior	3.62	0.40	0.084	0.219^**^	0.059	0.034	0.102	−0.056	0.181^*^	(0.884)

n = 276; ^*^p < 0.050, ^**^p < 0.010, ^***^p < 0.001. M = Mean, SD = Standard deviation; Gender: male = 1, female = 0; Job position: leader = 1, employee = 0.

### Confirmatory Factor Analysis

The confirmatory factor analysis is used to assess the factor structure of our measures. The hypothesized congeneric measurement model, in which abusive supervision, job performance, and employee innovation behavior loaded on separate factors, fit the data well [*χ*^2^ (186) = 554.661, *p* < 0.001, CFI = 0.947, NNFI = 0.926, RMSEA = 0.049, SRMR = 0.050]. This model achieves a better fit to the data compared to models that combine abusive supervision and job performance and employee innovation behavior into on factor [Two factor model: ∆*χ*^2^ (2) = 11.546, *p* < 0.01, CFI = 0.901, NNFI = 0.890, RMSEA = 0.073, SRMR = 0.075] and all of study variables into on factor [One factor model: ∆*χ*^2^ (3) = 985.876, *p* < 0.001, CFI = 0.654, NNFI = 0.611, RMSEA = 0.125, SRMR = 0.130]. These results support for the hypothesized measurement model. The above results are showed in Table 3.

**Table 3:** Results of confirmatory factor analysis.

Measurement model	*χ* ^2^	d.f	Δ*χ*^2^	CFI	NNFI	RMSEA	SRMR
Baseline model (Including abusive supervision、job performance、and innovation behavior)	554.661^***^	186	–	0.947	0.926	0.049	0.050
Two factor model (Including abusive supervision、job performance and innovation behavior combined to one factor)	563.690^***^	188	11.546^**^	0.901	0.890	0.073	0.075
One factor model (all of study variables combined to one factor)	1538.552^***^	189	985.876^***^	0.654	0.611	0.125	0.130

n = 276; ^**^p < 0.010 and ^***^p < 0.001.

### Hypothesis Tests

**Figure 2:**
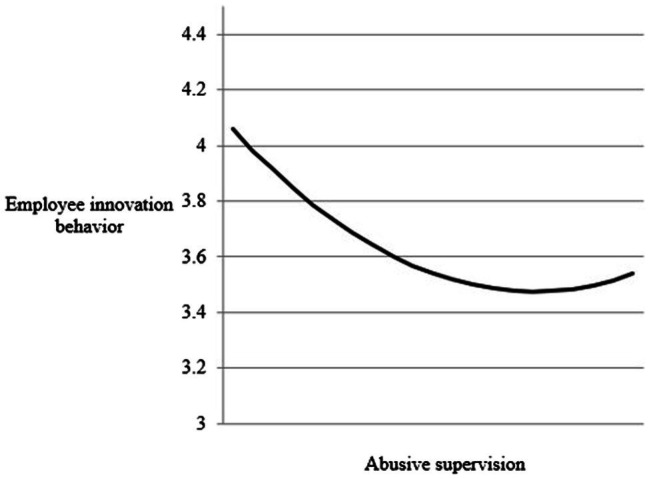
The pattern of non-linear relationship between abusive supervision and employee innovation behavior.

Table 1 shows the results of the hypothesis test. After controlling for gender, job position, length of employment, tenure working with supervisors, and negative emotion, the regression coefficient of abusive supervision is −0.161 (*β* = −0.161, *p* < 0.050) and the regression coefficient of the squared term of abusive supervision is 0.270 (*β* = 0.270, *p* < 0.010). That is, if abusive supervision is high or low, employee innovation behavior is better; while moderate abusive management, employee innovation behavior is at a low point, showing a U-shaped relationship (see Figure 2). The result is inconsistent with Hypothesis 1, which is not supported.

**Figure 3:**
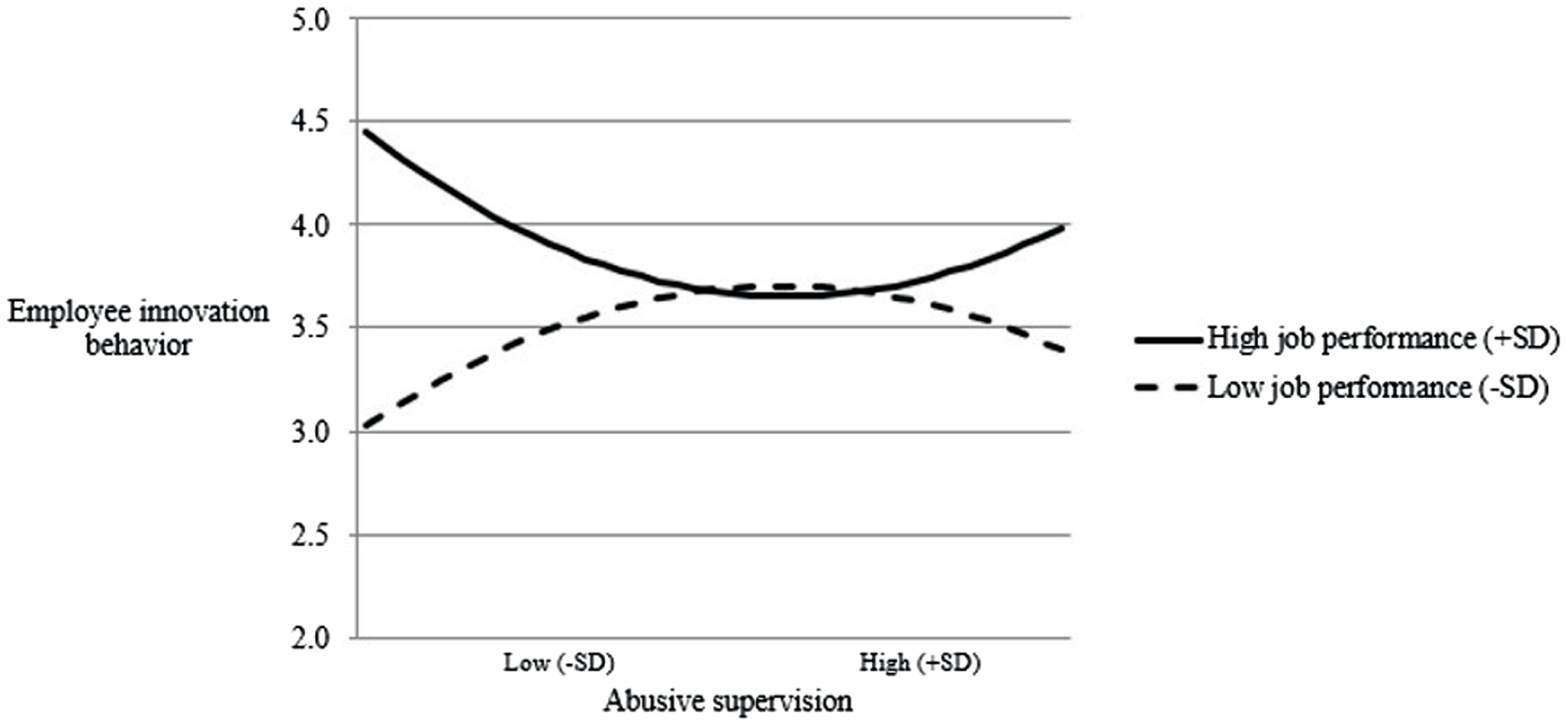
The moderating effect of job performance on non-linear relationship between abusive supervision and employee innovation behavior.

Next, while controlling for the product term of abusive supervision and job performance (*β* = −0.182, *p* < 0.050), the product term of squared term of abusive supervision and job performance also has a significant effect on employee innovation behavior (*β* = 0.338, *p* < 0.010). It can be initially concluded that job performance moderates the relationship between abusive supervision and employee innovation behavior. To further test the moderating effect of job performance, under the different levels of job performance a non-linear pattern of the relationship between abusive supervision and innovative behavior was developed (see Figure 3).

The results showed that for employees with high job performance (+ 1 SD), when they are subjected to moderately abusive management, they will inhibit their innovative behavior; and when they are subjected to severe supervisory abuse, they will increase their involvement in innovative behavior, showing a U-shaped curve relationship. Whereas, for employees with low job performance (− 1 SD) when they are subjected to moderately abusive management, they will promote their innovative behaviors; and when they are subjected to severe abusive supervision, they will reduce their innovative behaviors, showing an inverted U-shaped curve relationship. Thus, Hypothesis 2 is supported.

## Discussion

The study examines the relationship between abusive supervision, job performance, and employee innovative behavior based on the cognitive view of emotions (emotional cognitive theory and activation theory). Using a sample of frontline employees in the service industry as the research subjects, the study reveals a non-linear relationship between abusive management supervision and employee innovation behavior; and that job performance plays a moderating role in the non-linear relationship between abusive supervision and employee innovation behavior.

First, there is a non-linear relationship between abusive supervision and employee innovation behavior. However, this study found a U-shaped relationship between them: The level of employees’ innovative behavior is low when supervisors are moderately abusive. The Hypothesis 2 is not supported. Previous studies have showed that negative leadership behavior, abusive supervision, brings negative emotions, such as work tension, anxiety, and depression, to the subordinates and has a negative impact on employees’ innovative behavior (Yu et al., 2018). According to the cognitive theory of emotion, the key factor that elicits an individual’s emotional response is the perception of potential stressors (Smith et al., 1993; Huang et al., 2020; Liang et al., 2021). As the level of abusive management increases, employees are more likely to perceive it as a threat in their emotional appraisal, and thus are in negative emotions, such as helplessness and panic, making it difficult to devote additional enthusiasm and engagement to innovative behavior (Chen et al., 2021). In addition, the process of innovative behavior is highly uncertain and the results are not always satisfactory. Under abusive supervision, if the creative ideas of the employees are not supported by the supervisors, they may even be criticized and belittled, which will eventually lead the subordinates to be deterred from their work and become more conservative rather than risk-taking (Yang et al., 2019).

Besides, based on the strategic perspective, supervisors would promote their subordinates to accomplish performance goals that benefit the organization by intentionally expressing hostile behaviors (Shao and Mawritz, 2015). Thus, abusive supervision for employee development has a positive impact on employees’ innovative behavior. However, this finding for Hypothesis 1 is inconsistent with previous studies and may be attributed to employees’ job performance. Clearly, there is a U-curve relationship between abusive supervision and innovative behavior for employees with high level of job performance. Whereas, there is an inverted U-curve relationship between abusive supervision and innovative behavior for those with low level of job performance. This represents that Hypothesis 2 of moderating effect of job performance is supported.

The cognitive theory of emotion depicts that when employees react by associating and interpreting the behavioral or emotional displays of others, such as interpreting supervisory abuse as a lack of goodwill, jealousy, or hostility, it may cause employees to feel fearful and worry that they will be continuously mistreated by their supervisors in the workplace (Chan and Drasgow, 2001; Liang et al., 2021) and to reduce their involvement in innovation. However, as the level of abuse deepens, supervisors’ jealousy will also change the emotional experience of the subordinates, and the more the subordinates feel frustrated with the supervisor (Cohen-Charash and Mueller, 2007). At this point, competent employees anticipate their own possibilities of achieving their innovative goals and transform the stimulus into energy and then increase their innovative behavior in order to improve their chances of promotion and avoid the persistent unfriendly treatment by their supervisors.

On the contrary, when employees perform poorly, their motivation to innovate is inhibited (Lord and Levy, 1994). Activation theory depicts that moderate stress motivates employees while depleting resources and brings more possibilities for success (LePine et al., 2016). The moderate level of abusive supervision stimulates employees to generate moderate job stress, under the guilt and frustration generated by those performing poorly, to improve the evaluation of supervisors’ abuse as a spur to activate innovative thinking (Ingold et al., 2015). When the abuse is low, it is difficult to stimulate subordinates to engage in innovative behavior in low-pressure work situations. When the abuse is high, it is more likely to cause low-performing subordinates to make mistakes at work and inhibit their innovative behavior because of the pressure going beyond their psychological tolerance.

## Theoretical and Practical Implications

The study found that the moderating effect of job performance on the non-linear relationship between abuse management and employee innovative behavior. Employee innovative behavior is related to the long-term development of the organization, but the process of innovation requires continuous exploration, experimentation, and improvement, and the results are unpredictable (Yang et al., 2019). Therefore, the leadership behavior may deeply affect employees’ willingness to innovate in the face of the great risks brought by innovation. The question of how to encourage and promote employees to actively engage in innovation is one that leaders must face and think about.

First, based on the cognitive theory of emotion, abusive supervision is like a threat to the supervisor’s evaluation of his subordinates, which will have a negative impact on their psychological and work behaviors, such as increasing the psychological burden at work, triggering emotional response of unfair perceptions and dissatisfaction, and avoiding the innovation process or no longer investing time and energy in innovation (Van den Bos et al., 2008; Yang et al., 2019). However, some studies indicated that the moderate level of abuse helps to arouse employees’ innovation (Shum et al., 2014). Therefore, managers should recognize the possible benefits and harms of abusive supervision for their employees, adopt abusive behavior as a leadership style cautiously according to their employees’ performance and stress resistance, and tolerate employees’ innovation failures and give them flexibility for trial and error.

Moreover, managers should effectively regulate their own emotions and behaviors at work. For example, when a team suffers a major setback or an emergency occurs, managers should not feel free to take out their emotions on employees, shirk their responsibilities, or even make hostile verbal or non-verbal behaviors toward their subordinates. Therefore, when recruiting and selecting managers, organizations need to assess their leadership style and performance under high pressure, especially abusive or negative supervision. Then, leadership training should be developed for managers to have a comprehensive understanding of the impact of abusive supervision on work-related outcomes, so that they can avoid or improve the negative effects of supervisors’ abuse on employees’ innovative behavior.

Finally, job performance can moderate the effect of abuse supervision on employees’ innovative behavior. Employees’ job performance reflects the results of past behaviors, and high performance means that they are competent, confident, efficient, and show a higher sense of control and satisfaction with their work when facing creative work (Mitchell and Ambrose, 2012). However, in response to different levels of abusive supervision, employees who perform well and those who perform poorly show opposite results in their innovative behaviors. The study reveals that when supervisors should perform different level of abusive behaviors depending on employees’ job performance, so that employees can devote themselves to innovative behaviors with more enthusiasm and engagement for work.

## Limitations and Directions for Future Research

This study found that job performance has a moderating effect on the non-linear relationship between abuse supervision and employee innovation behavior but there are some limitations that need to be further studied in future research. First, Although the questionnaire survey was conducted at two points in time and used employee’s and supervisor’s assessment, the questionnaire content related to negative leadership behavior—abusive supervision—may have a social desirability effect. Nevertheless, the results of mean (1.51) and standard deviation (0.59) of abusive supervision is low in this study, which is consistent with previous studies (e.g., Wang et al., 2019; Zhu and Zhang, 2019). And there is a low correlation coefficient among key variables (abusive supervision, job performance, and innovative behavior). Therefore, the common method variance is relatively low in this study. Future research in the measurement of work-related outcome variables, such as job performance, could use objective data to reduce the effects of methodological error (Podsakoff et al., 2003, 2012).

Second, this study used a cross-sectional research method and therefore could not verify the causal relationship. Future research should adopt a longitudinal approach to identify the causal relationship between abusive supervision, job performance, and employee innovation behavior.

Finally, the study showed that employees’ job performance moderates the non-linear relationship between abusive supervision and their innovative behavior. However, the mechanism of the influence and effect of abusive supervision on employees’ innovative behavior is complex, and there is no consistent consensus in the field of these studies. There may be other mediating or moderating variables in this relationship, such as jealousy (Lee and Duffy, 2018; Yu et al., 2018) and fairness (Yang et al., 2019). Future research can explore the linear or non-linear relationships between supervisors’ abuse and innovative behavior or other outcome variables from different perspectives, as well as the mechanisms between them.

## Conclusion

This study investigated 276 frontline employees from the service industry in China to explore the mechanism of the non-linear relationship between abusive supervision and employee innovation behavior. Based on the cognitive view of emotions, it is proposed that job performance is the key moderating variable affecting this relationship. Specifically, an inverted U-shaped relationship between abusive supervision and employee innovation behavior was found when employees’ job performance was low, and a U-shaped relationship was found when employees’ job performance was high. This study provides theoretical and practical recommendations for managers to use appropriate negative leadership behaviors “abusive supervision” to promote employee innovation in their workplace.

## Data Availability Statement

The raw data supporting the conclusions of this article will be made available by the authors, without undue reservation.

## Author Contributions

K-SY main contribution is in literature and theory. T-JW is in charge of research methodology and survey. TN is in charge of statistical analysis. All authors contributed to the article and approved the submitted version.

## Funding

Funding was provided by Huaqiao University’s Academic Project Supported by the Fundamental Research Funds for the Central Universities (20SKGC-QT02).

## Conflict of Interest

The authors declare that the research was conducted in the absence of any commercial or financial relationships that could be construed as a potential conflict of interest.

## Publisher’s Note

All claims expressed in this article are solely those of the authors and do not necessarily represent those of their affiliated organizations, or those of the publisher, the editors and the reviewers. Any product that may be evaluated in this article, or claim that may be made by its manufacturer, is not guaranteed or endorsed by the publisher.
